# Plant‐Derived Natural Products and Their Nano Transformation: A Sustainable Option Towards Desert Locust Infestations

**DOI:** 10.1002/open.202400271

**Published:** 2024-11-26

**Authors:** Patrick Mangundu, Rebaone Makaudi, Hugues Kamdem Paumo, Bathabile Ramalapa, Lesego Tshweu, Naledi Raleie, Lebogang Katata‐Seru

**Affiliations:** ^1^ Department of Chemistry School of Physical and Chemical Sciences North-West University Private Bag X2046, 2735 Mmabatho South Africa; ^2^ Biotherapeutics Delivery laboratory Centre for Nanostructures and Advanced Materials Council for Scientific and Industrial Research (CSIR) Pretoria 0001 South Africa

**Keywords:** Plant-derived insecticides, Desert locust, Nanotechnology, Nano-formulations, Pest control

## Abstract

The desert locust has been recognized as the most devastating migratory pest in the world. Swarms of this pest have been threatening vast regions of pastures and crops in Africa, Middle East, and South Asia. The biological management of expanding swarms has become a strategy of particular interest due to environmental awareness and economic issues associated with chemical pesticides. The present review aims to explore the latest updates and information about pesticidal plants that are distributed across Africa. Searches on Web of Science, Google Scholar, PubMed, and Scopus databases from 2013–2024 revealed a total of 22 plant species probed for insecticidal activities against desert locusts. The formulation, active ingredients, and biological effects of essential oils and other extracts from these plants are presented. Despite the promising anti‐insecticidal effects of the plant extracts and compounds, issues related to their solubility and instability under environmental conditions have been observed. To address such major quality defects, methods for the encapsulation of plant natural products within nanostructures are detailed. Given the presence of bioactive compounds with nucleophiles bearing functional groups, the reported plant extracts have been exploited to fabricate metal nanoparticles with inherent insecticidal activities. In this paper, a holistic overview of prepared phytochemical‐coated metal nanopesticides is also presented. In summary, this study offers insights into the integration of nanoformulated natural resources as a more sustainable option to control desert locust invasions.

## Graphical abstract

The review explores the latest updates (2013–2024) and information about pesticidal plants (22 plant species) that are distributed across Africa and offers insights into the integration of nanoformulated natural compounds as a more sustainable option to control desert locust invasions.

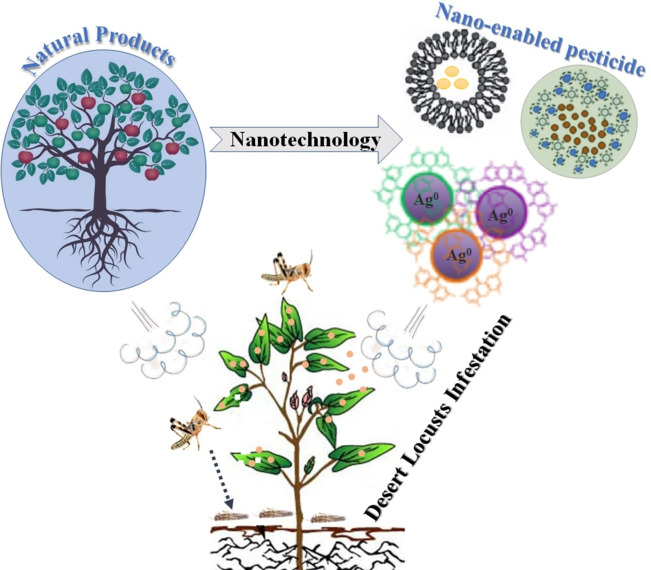

## 1 Introduction

**Figure 1:**
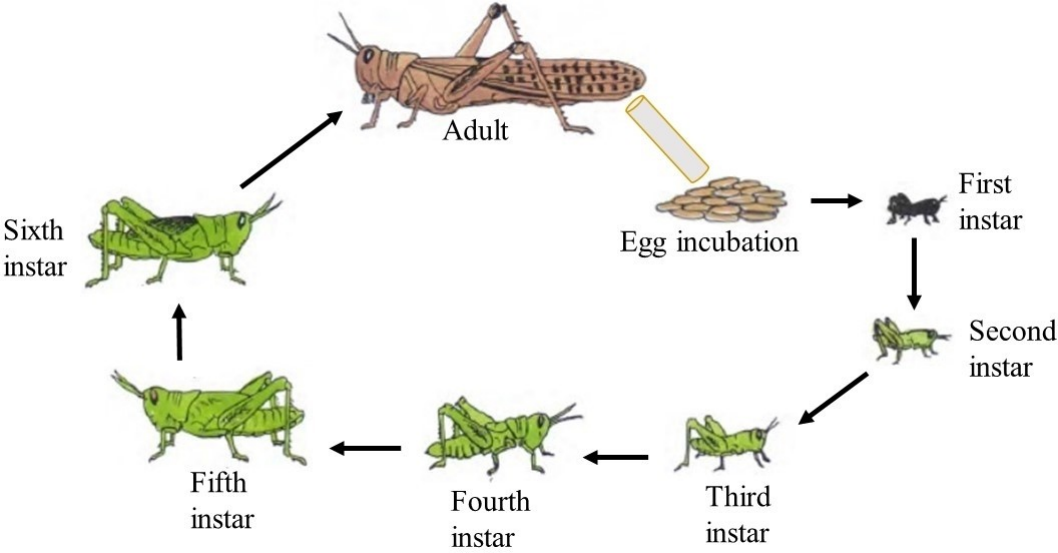
Life cycle of the most destructive migratory pest in the world (*S. gregaria*).

Locust plagues and surges have been prevalent since time immemorial, but current outbreaks are mostly attributed to anthropogenic climate change.^[28]^ Notable incidents have occurred in various regions of Africa, such as East Africa in 2020 and South Africa in 2022.[^94^, ^96^] These events, coupled with the significant influence of warming in the Indian Ocean, highlight that climate change's repercussions extend beyond rising global temperatures, leading to unprecedented and catastrophic natural hazards. Locusts typically exhibit phase polyphenism and undergo behavioral changes based on population density throughout their life cycle. They transition between the solitary and gregarious stages. During periods of low population density, locusts tend to be solitary. However, under crowded conditions, these short‐horned grasshoppers form large colonies and engage in mass migration. Extensive research has established that desert locusts, specifically *Schistocerca gregaria* (Forskål, 1775), are the most migratory and destructive pests worldwide.^[48]^ The desert locust is one of the nineteen known species of locusts found globally.^[52]^ There are three stages in their general life cycle: egg, nymph (first to sixth instar), and adult (Figure 1).

The desert locust's phase change involves 532 genes among which 90 are differentially methylated in the solitary form as opposed to the gregarious form.[^24^, ^33^] In both forms, common features include the ability to transmit their phase to the progeny and revert to the alternative phase under appropriate conditions.^[29]^ In the transition phase, there are significant changes in the amounts of neurotransmitters, alterations in neurons, especially in synaptic morphology secreted in the nervous system, leading to crowding.[^17^, ^24^] In fact, an experimental injection of serotonin (neurotransmitter) or its analogs induces transition from solitary to the gregarious forms.^[24]^ Furthermore, several behavioral activities, such as migratory flying, settling on the ground for basking, feeding, and resting during the daytime, and roosting on plants at night are generally observed in *S. gregaria* swarms (Uvarov, 1977).

These pests reproduce rapidly and are adapted to live in arid and semi‐arid habitats that are found in the Northern, Eastern, and Southern parts of Africa. These regions experience unusual precipitations annually. Warmer soil temperatures (15–35 °C) and heavy rainfall create favourable environmental conditions for the breeding of desert locusts.[^96^, ^107^] Warm winters with temperatures varying between 25–32 °C coupled with humidity 85–92 % also favour locust development. This leads to the formation of swarms and a change in behavior that forces the pests to migrate and voraciously feed on almost all vegetation, including key food crops.^[86]^

**Figure 2:**
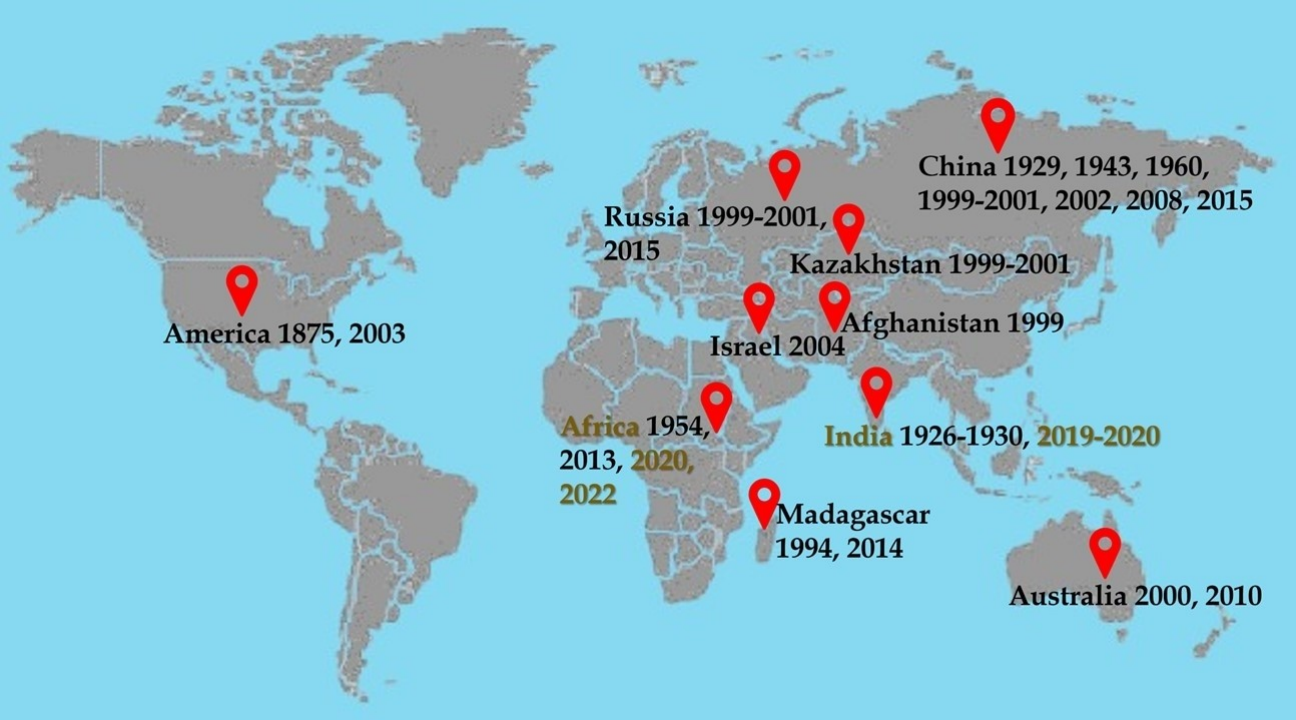
Historical episodes of locust outbreaks in the past 150 years.

*Schistocerca gregaria* plagues represent a serious threat to agricultural production, rural livelihood, and food security in Africa. The worst incursion reported in decades occurred in East Africa (Djibouti, Eritrea, Ethiopia, Kenya, Somalia, Sudan, Tanzania, and Uganda) in 2020 under the acute pressure of COVID‐19 pandemic.^[109]^ According to the Food and Agriculture Organization, this pest upsurge led to >30 % cereal harvest loss, thereby driving nearly 25 million people into severe food insecurity in Africa (FAO 2020). Localised swarms that ravage crops and pastures have also affected several countries in southern Africa. For example, swarms of locusts have lately been spotted in three provinces of South Africa, *vis*. Northern Cape, Eastern Cape, and Western Cape, causing a stream of agricultural damage. Locust plagues in the Southern region of Africa are reported to emerge from Nama‐Karoo,[^26^, ^91^] a semi‐arid inland biome of Namibia and South Africa. In this ecoregion, precipitation ranges from approximately 100 to 400 mm per annum, although rainfall is unpredictable and prolonged drought is common. The major locust events from 1875 to date are presented in Figure 2. Notably, the most recent and pronounced invasions were recorded in India and Africa.

The resurgence of desert locust swarms in Africa over the last two years poses an unprecedented risk of food crisis in already struggling economies. For this reason, news bulletins and updates on the locust situation are published by the FAO on a regular basis. This allows for early intervention against the confined outbreaks before plagues arise. Chemical control is often adopted to keep the pest population below the density that triggers aggregation into swarms. However synthetic pesticides have been reported to demonstrate adverse impact. Chemical pest control agents are likely to contaminate freshwater systems and food chains, proving detrimental to human health.^[96]^

Clearly, emergent countries have very little financial resources and encouraging research and development into inexpensive, safer, and efficient strategies to prevent *S. gregaria* plagues can be an investment of guaranteed returns. Growing interest in this regard has led to the development of biological control strategy using plant extracts. Nonetheless, natural products are prone to rapid degradation in the environment. Nanotechnology has paved the way for the advancement of plant extracts derivatives with improved stability and efficacy. The present work aims to summarize up‐to‐date information regarding the use of nanoformulations using pesticidal natural products from plants, for preventive control of desert locust swarms. By offering a comprehensive review of nanotechnology‐based natural pesticides, this paper is expected to pave the way for future advances in the development of safer and efficient strategies for pest management with the use of biopesticides.

## 2 Methods to Prevent Outbreaks of Desert Locusts

Numerous cultural mechanical practices have been employed to curb the upsurges of desert locusts and their damage to crops. These entail the use of farm operations such as harrowing, herding, tilling, burning pastures, and trampling to destroy and collect the insects.^[106]^ In Africa, desert locusts are frequently used in a variety of dishes due to their nutritional value. A recent report by Kinyuru^[63]^ on the nutritional details of *S. gregaria* revealed that 100 g contains 46 g of protein, 32 g of fat, 208 mg of calcium, and 450 Kcal energy. Although entomophagy is a mitigation approach that can contribute to restraining plagues evolution, the physical control measures employed in the management of desert locusts are labour intensive, time consuming, and habitually ineffective. Clouds of smoke, high‐frequency sound, and fire are also argued to keep the desert locusts at bay. However, these mechanical techniques are not sustainable owing to environmental air and noise pollution.

**Figure 3:**
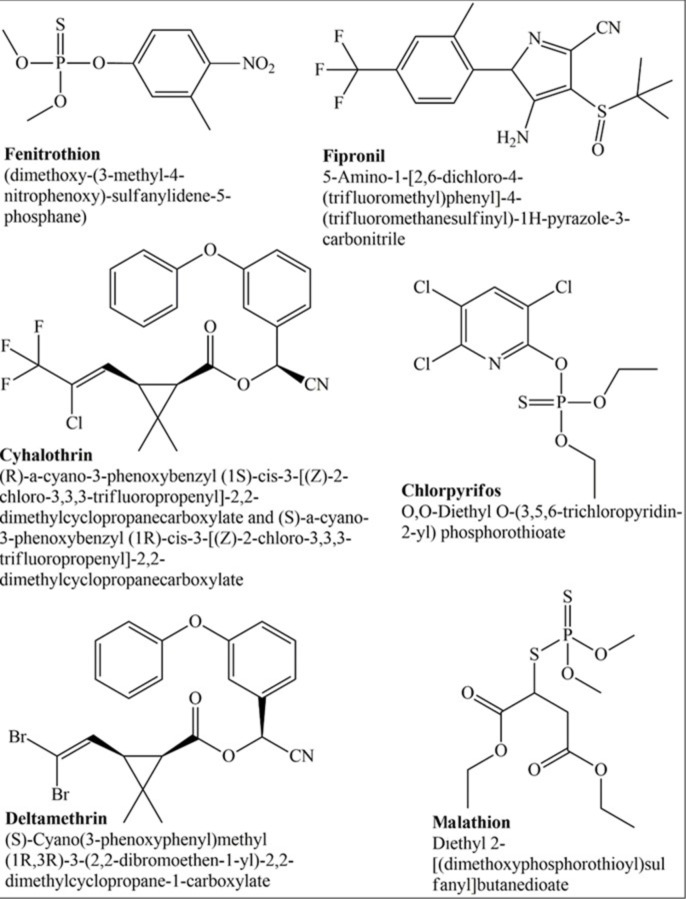
Chemical structures of commonly used synthetic pesticides.

Contact application of concentrated chemical formulations onto locusts remains the most effective control method.^[69]^ This standard practice is accomplished using hand‐held sprayers if swarms are of lesser extent. In the case of flare‐ups, vehicle‐ and/or aircraft‐mounted sprayers are required to suppress *S. gregaria* populations. Fenitrothion, deltamethrin, chlorpyrifos, malathion, fipronil, and cyhalothrin are the common bioactive chemicals championed for locust control (Figure 3). These nerve agents are laudable but tend to be non‐selective in action, thereby posing a noteworthy threat to non‐target microorganisms.^[102]^ Moreover, the pest control agents are likely to contaminate the freshwater systems and food chains. In 2021, the U.S. Environmental Protection Agency asserted it decision to end the use of chlorpyrifos on all food products.^[76]^ The adverse impacts of residues of synthetic pesticides to human health are well‐reviewed in the literature.[^48^, ^96^] Exposure to synthetic pesticides has also been reported as detrimental to edible crops. For example, research findings from the physiological response of tomato (*Solanum lycopersicum* L.) seeds exposed to imidacloprid, mancozeb, and diazinon revealed low germination rate, disrupted photosynthesis, and morphological deformation during plant growth.^[52]^ Decreasing chemical residue concentration after a large‐scale control of desert locust with chemical pesticides is always a major concern. Cost, safety, and resistance are among the factors that influence the use of a pesticide on a large scale. The development of resistance to a pesticide is the normal response of an insect population to the selection pressures applied by the continuous use of insecticides. Nevertheless, it has not been confirmed whether a naturally occurring population of the desert locust has ever been exposed to pesticides over several generations.

## 3 Biological Control Methods Based on Plant Phytochemicals

Because of growing concerns over the application of synthetic compounds, naturally occurring active agents have been persuasively tested by the scientific community as alternative locust control insecticides. These include the use of entomopathogenic microorganisms and plant extracts[^38^, ^76^, ^105^] The biopesticides offer less hazardous ways to control an age‐old problem of desert locust infestations. The control of pests using extracts from selected plant species is a method that was implemented earlier before the green revolution and the synthesis of highly effective pesticides and their large‐scale use.^[68]^ Plant extracts with reported insecticidal activity consist of a wide variety of bioactive compounds. These include alkaloids, amino acids, flavonoids, limonoids, polyphenols, terpenoids, saponins, and glucosides. For example, alkaloids anatabine, anabasine, and nicotine obtained from *Nicotiana tabacum* (Tobacco) infusion, have a long tradition to serve as neurotoxins for pest control. These biomolecules mimic the excitatory mediator acetylcholine in the central nervous system of the pests to incite symptoms of poisoning.^[32]^ Another plant employed as a source of compounds with growth‐disrupting ability against pests is *Azadirachta indica* (also known as neem). Bark, leaf, and seed extracts of this tree have been argued to act as biological pesticides to control *S. gregaria*. For example, treatment of crowded fifth nymphal instars of *S. gregaria* using azadirachtin (2–121 μg/g), a limonoid accounting for >30 % mass of neem seeds, led to delayed moult and death after 20 days.^[12]^ Neem tree is widespread across the Sahel, and it is advocated as a useful plant in the treatment of malaria. The antifeeding activity of *A. indica* against desert locusts has also been described in the literature. Fourth instar larvae of *S. gregaria* were found to suffer from weight loss after feeding on clover leaves treated with azadirachtin (5, 10, 15, 20 and 25 % in acetone). 100 % mortality rate was achieved after 72 h exposure to 25 % azadirachtin‐treated leaves.^[13]^ Similar observations were noted by Abdelbagi and coworkers (2019) for second, third, fourth and fifth instars in contact with pearl millet plants treated with varying concentrations (1, 5, 10 and 20 % w/v water) of aqueous extract of neem seeds. Opiyo,^[81]^ in a study on the pesticidal efficacy of crude extracts of *Ocimum suave* and isolated compounds, highlighted the significantly better repellent activity of the former, thereby suggesting a likely synergistic effect in phytochemicals action.

The organic extractions of *Fagonia bruguieri* were reported by Ghoneim et al.^[46]^ for pronounced inhibitory action against *S. gregaria*. A decreased acetylcholinesterase activity was recorded in the haemolymph of adult population treated with butanol and petroleum ether extracts (15 and 30 %). Inactivation of acetylcholinesterase enzyme leads to accumulation of acetylcholine, disruption of neurotransmission, and eventually paralysis. *Calotropis procera* is a perennial shrub native to Asia and several subregions in Africa (North and tropical Africa). Habitually, this plant is being used for fuelwood and the treatment of constipation, muscular spasm, and fever.^[75]^ However, Kaidi et al.^[59]^ also highlighted the insecticidal effect of *C. procera* extract against *S. gregaria*. Acetone leaf extract of *C. procera* (0.5 g/mL) was found to induce antifeedant effect on adult desert locusts and a remarkably high mortality rate (100 %) after 7 days ingestion. Another species of *Calotropis* that has shown antifeedant potency against desert locusts is *C. gigantea*.[^14^, ^83^] Extraction of the root bark of this plant in methanol afforded an amino acid with a substantial feeding deterrent effect (>80 %) on fifth instar nymphs. 16 % larvae failing to complete ecdysis, 46 % adults’ mortality, and 48 % abnormally formed adults were recorded by Mahdy et al. after 24 h feeding on leaves treated with petroleum ether extract of *Oryza sativa* bran (20×10^3^ ppm).^[70]^ Failure of ecdysis sequence and disintegration of the gut epithelium cells were also observed in fifth instar larvae and adults treated (contact or ingestion) in the presence of ethanol extract of *Schouwia purpurea*.^[74]^

According to literature information, plant essential oils, which were obtained by hydro‐distillation have also been screened for insecticidal activity against *S. gregaria*. The essential oil of *Cleome arabica* leaf, a drought tolerant plant widely distributed in North Africa, has been reported to display median lethal time of 9 and 41 min against fifth stage larvae and adults, respectively.^[61]^ Seed oil from this plant has also been found to severely affect fifth instar male larvae (Mortality: 91 % after 16 days ingestion) than female.^[9]^ Within the same context, oil extracted from *Peganum harmala* was found to be relatively more potent (lethal time 6 and 19 min against larvae and adults). By using third instar nymphs as model, the essential oil obtained from seeds of *Cuminum cyminum* (well‐known as cumin) achieved acute toxicity (Mortality 91 %) at 55 μg/g after 24 h contact.^[71]^ Furthermore, the complete dissipation of this toxic activity was experienced after 12 days, evocative of the ineffectiveness of phytochemicals over time. Moreover, it is important to note that the biological activity of essential oils is usually not ascribed to any single phytochemical that stands out against the desert locusts.^[61]^ Presumably, minor/major phytoconstituents interact in a complex and unforeseen manner to achieve the biological activity and minimize a potential pest resistance. A mixture of three essential oils from different plant materials, namely *Citrus aurantium dulcis* (orange peel), *Gaultheria procumbens* (wintergreen), and *Carum carvi* (Caraway) were described as commendable insecticide to control *S. gregaria* population.^[2]^ The synergistic effect of these extracts complemented by sodium bicarbonate (47 %) resulted in 80–100 % mortality after 24 h. Interestingly, no adverse effect on the growth of wheat seedlings was observed when applying this formulation.

In the past decade, numerous studies provided sufficient evidence that a selection of plant extracts from leaves, seeds, roots, and/or stem could serve as efficacious biological control candidates against the desert locusts. In this respect, Table 1 depicts the geographic distribution, formulation, active ingredients, and biological effects of 22 plant species distributed in Africa and reported to demonstrate activity as potential biopesticides between 2013 and 2023. Nevertheless, key drawbacks associated with the practical applications of the biopesticides derived from plant materials encompass a short shelf‐life and decreasing efficacy over time due to greater sensitivity to environmental factors, and the use of organic solvents for improved solubility and ideal formulation. This has led to the search of natotechnology‐based strategies to enhance the stability and improve the overall efficacy of plant‐derived biopesticides.

**Table 1:** Reported plant materials and their active ingredients as biopesticides against desert locust.

	Species (Families)	Geographic distribution	Formulation	Active ingredients	Biological effects	References
1	*Azadirachta indica* L. (Meliaceae)	Native to the Indian subcontinent and introduced in most countries in West and Central Africa.	Seed aqueous extract (soaking for 24 h at room temperature). The filtrate was used as formulation.	Azadirachtin	Interrupts the production of ecdysone, a hormone that stimulates metamorphosis.	^[5]^
			Root methanol extract formulated in ethanol	Azadirachtin	Antifeedant activity	^[100]^
2	*Fagonia bruguieri* DC. (Zygophyllaceae)	Distributed in Asia, South America, and North Africa.	Petroleum ether and butanol extracts of the aerial parts (maceration)	Not reported	Interferes with the neurotransmitter signaling.	^[46]^
3	*Calotropris procera* (Apocynaceae)	North Africa, Tropical Africa, South Asia, and Western Asia	Leaf acetone extract (maceration), used as formulation. Water and ethanol extracts of the aerial parts (Soxhlet method), formulated in distilled water.	Alkaloids	Disintegrates the cells of the gut epithelium and causes a decline in food ingestion.	[^59^, ^74^]
4	*Schouwia purpurea* subsp. (Brassicaceae)	North Africa, West Africa, East Africa, and Asia	Ethanol extract of the aerial parts (soxhlet method), formulated in distilled water.	Not reported	Causes failure of ecdysis sequence and alteration of the intestinal epithelial cells.	^[74]^
5	*Zizyphus lotus* L. (Rhamnaceae)	Mediterranean region				
6	*Ammi visnaga* L. (Apiaceae)	Native to Egypt and distributed in Europe and over the Mediterranean region	Ethanol extract of the fruits (maceration), formulated in ethanol, butanol, and petroleum ether.	Khellin, visnagin, and pyranocoumarin	Impairs the hormonal regulation and inhibits metamorphosis.	^[44]^
7	*Punica granatum* L. (Lythraceae)	Native to Persia and cultivated the Mediterranean region and South Africa	Peel ethanol extract (maceration), formulated ethanol, butanol, and petroleum ether.	Not reported	Disturbs the hormonal control of metamorphosis.	^[45]^
8	*Nigella sativa* L. (Ranunculaceae)	Native to North Africa, Asia, and Europe	Seed methanol extract, formulated in methanol, butanol, and petroleum ether.	Not reported	Interferes with the developmental transitions.	^[47]^
9	*Oryza sativa* L. (Poaceae)	Native to China and domesticated in several regions of the world, including Africa	Bran petroleum ether extract	Not reported	Affects the development of larvae by causing inhibition of moulting.	^[70]^
10	*Citrullus colocynthis* L. (Cucurbitaceae)	Native to the Mediterranean basin and Asia	Hexane and methylene chloride extracts (soxhlet method), formulated in a mixture of butanol and xylene	Saponins	Instigates the epidermal detachment and the appearance of cytoplasmic vacuoles.	^[77]^
11	*Allium cepa* L. (Amaryllidaceae)	Native to Asia and introduced worldwide	Leaf essential oil (hydrodistillation)	p‐Cymene (40 %) and disulfide dipropyl (21 %)	Causes a decrease in digestibility and interferes with the structure of the rectal pads.	[^25^, ^71^, ^97^] Mansour et al., 2015
12	*Matricaria chamomilla* L. (Asteraceae)	Native to Europe and introduced in Asia, America, Australia, New Zealand, and North America	Essential oil of the whole plant (hydrodistillation)	β‐Franesene (31 %), germacrene‐D, bicyclogermacrene, and d‐cadunene		
13	*Pelargonium radula* (Geraniaceae)	Native to South Africa	Essential oil of the whole plant (hydrodistillation)	Cetronellol (23 %), geranial (14 %), linalool (8 %), iso‐menthone (5 %)		
14	*Ocimum basilicum* L. (Lamiaceae)	Native to Asia and Tropical Africa	Essential oil of the whole plant (hydrodistillation)	Linalool (51 %), eugenol (16 %), 1,8 cineole (6 %), and cadinol		
15	*Cuminum cyminum* L. (Apiaceae)	Native to the Mediterranean region and introduced in the Middle East, China, and India	Seeds essential oil (hydrodistillation)	Cuminic aldehyde (21 %), α‐terpinene (19 %), p‐mentha‐1,3 diene‐7‐al (16 %), β‐pinene (15 %), β‐cymene (5 %)		
16	*Origanum vulgare* L. (Lamiaceae)	Originating from the Mediterranean region and distributed in Europe and Asia	Essential oil of the whole plant (hydrodistillation)	β‐Terpinene‐4‐ol (23 %) β‐Terpinene (15 %), Sabinene‐Phelandrene (7 %)		
17	*Jatropha curcas* L. (Euphorbiaceae)	Native to North, Central and South America, and introduced in South Africa and Asia	Seed essential oil using hexane as solvent (soxhlet process)	Not reported	Reduces the fecundity of female species and causes malformation.	^[21]^
18	*Lepidium sativum* L. (Brassicaceae)	Native to West Asia and Egypt, and introduced all over the word	Essential oil (purchased)	Glucosinolates	Alters the acetylcholinesterase activity and promotes the unsuccessful moulting.	^[6]^
19	*Datura stramonium* L. (Solanaceae)	Introduced in North and East Africa	Seed essential oil using hexane as solvent (soxhlet method)	Scopolamine and hyoscyamine	Causes abdominal distension and inhibits the rectal reabsorption of Water.	^[8]^
20	*Cleome arabica* L. (Capparaceae)	North Africa, East Africa, Middle East	Leaf essential oil (hydrodistillation) Seed essential oil using hexane as solvent (Soxhlet method)	Triterpenoids, flavonoids, anthraquinone, saponins, tannins, steroids, glycosides, and alkaloids	Cause balance disorders after a temporary increase in acetylcholinesterase enzymatic activity. Interferes with the physiological processes that regulate water loss.	[^9^, ^61^]
21	*Peganum harmala* L. (Zygophyllaceae)					
22	*Nerium oleander* L. (Apocynaceae)	Native to the Mediterranean Basin and cultivated worldwide	Fresh leaves	Oleanderocioic acid, quercetin, kaempferol, oleandrin, and oleandrigenin	Causes a dysfunction of the nervous system and a slowdown of locomotion.	[^18^, ^37^]

## 4 Nanoencapsulation of Plant‐Derived Pesticides

**Figure 4:**
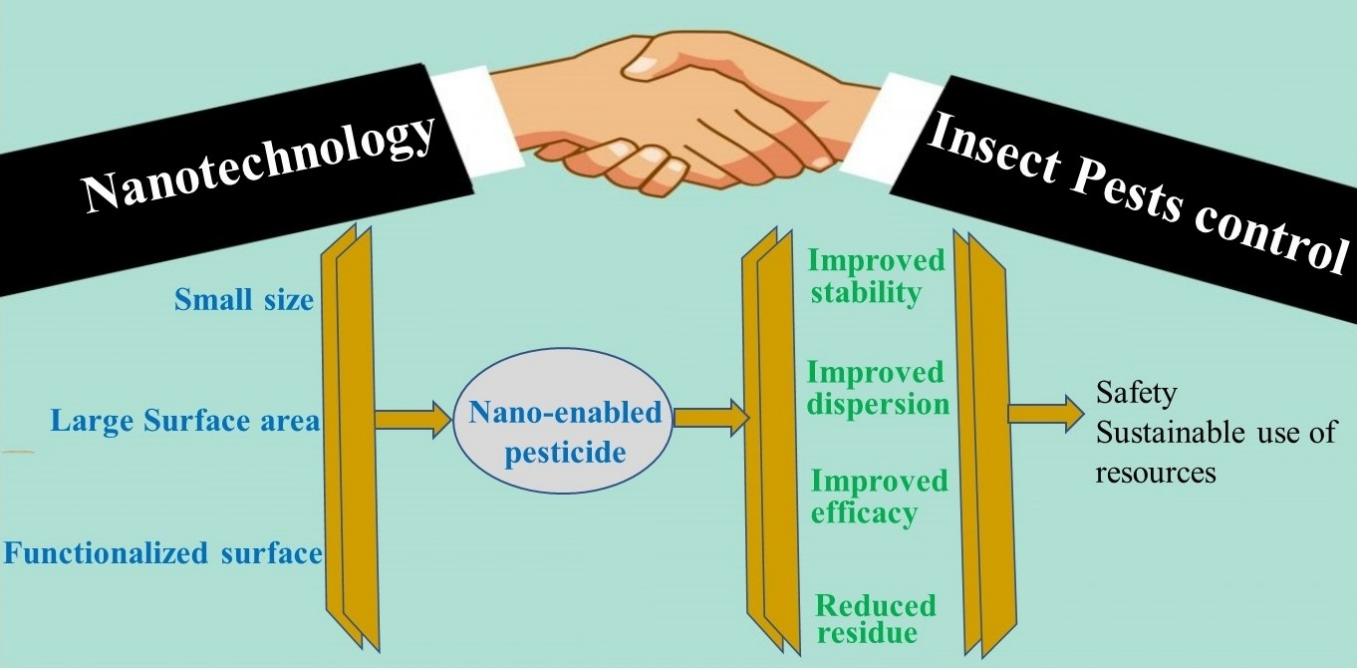
Properties of nano‐enabled pesticides and their benefits.

Several aspects including application technique, doses, environmental conditions, and physicochemical properties of the insecticides influence the effectiveness and efficiency of control measures. Indeed, >90 % of the applied control agents either do not reach the target or are not stable enough to achieve effective crop protection.^[88]^ The potential applications of nanotechnology in agriculture include seed priming, monitoring devices, and agrochemical delivery systems.^[30]^ The use of nanotechnology in the biological control of insect pests remains underexplored,^[80]^ despite the great prospect in revolutionizing the agricultural sector. Relative to bulk materials, nanoengineered materials are characterised by particles with ultra‐fine size, larger surface area/unit volume, and target tunable properties. These can be exploited to extend the durability of pesticidal phytochemicals in the environment, limit their volatility, and improve their increased systemic activity. The benefits of nano‐enabled botanical pesticides over the conventional products may also include better dispersion in water and reduced application rates (Figure 4).

The development of natural pesticides that can withstand the deteriorating conditions serve as a concept to achieve formulations with enhanced activity and field performance. Phytochemicals consist of sensitive compounds that are unstable under hostile environmental conditions, such as UV radiation, light intensity, heat, pH, and ambient oxygen/air oxidation. Poor bioavailability, solubility and absorption, and high instability are frequently observed with biologically active plant natural products, as opposed to their synthetic counterparts.^[110]^

**Figure 5:**
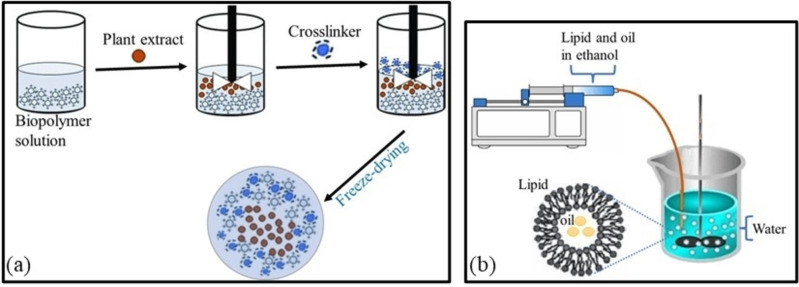
Nanoencapsulation of plant extract by (a) ionotropic gelation and (b) ethanol injection.

The encapsulation of phytochemicals in suitable nanostructures is a strategy that allows for protection of the active compounds from the adverse environmental aspects and improves the insecticidal value of plant extracts. For example, the encapsulation of essential oil of *O. basilicum* in chitosan‐tripolyphosphate (TPP) nanoparticles by the ionotropic gelation method afforded spherical‐like nanostructures (198–373 nm) with enhanced biological activity, as compared with the oil formulation in a free form.^[25]^ The as‐prepared nanoparticles displayed high encapsulation efficiency (EE) of 75 %. Electrostatic phenomena between the positively charged groups on chitosan (−NH_3_^+^) and counterions tripolyphosphate engender nanoparticles under mechanical stirring as illustrated in Figure 5(a). This method has also been carried out using negatively charged −COO^−^ groups on alginate and counter ions Ca^2+^ to entrap essential oil of *A. indica*.^[95]^ Particles obtained through a stirring mixture of alginate 2 % and oil extract 10 mL in the presence of CaCl_2_ as a crosslinking reagent, enabled a slow release of phytochemicals (7 %) in neutral milieu (pH 6.5) after 1 h. This release behaviour can be important to achieve zero‐toxicity to non‐targeted organisms. The presence of amino and carboxyl groups in chitosan and alginate biopolymers makes their derived nanoparticles pH responsive. Under acidic conditions, basic functional groups can be responsible for swelling and quick release of phytochemicals encapsulated into chitosan nanoparticles. Conversely, protonation of the carboxyl groups of alginate‐based nanoparticles in acidic milieu can lead to less pronounced electrostatic repulsion between these functional groups, shrinkage of the nanocarrier, and ultimately no release of entrapped phytochemicals.

Liposomal nanoformulation is another technique that can also achieve high encapsulation of phytochemicals in nanocarriers[^40^, ^43^] Lipid‐based nanoparticles can be utilised to facilitate the dispersion of essential oil in water and ease attachment/absorption of the bioactive compounds through the insect cuticle.^[85]^ The successful encapsulation of essential oil from *O. vulgare* into nanoliposomes has been described by Kryeziu and coworkers (2022) using the ethanol injection procedure (Figure 5b). A mixture of lipoid S100, cholesterol, and *O. vulgare* oil in ethanol was injected in water to give particles of average size (AS) 319 nm and EE of 85 %. Interactions between the functional groups on the carrier material and the hydroxyl groups of phytochemicals increase the encapsulation efficiency (Aguilar‐Pérez et al., 2021).

**Figure 6:**
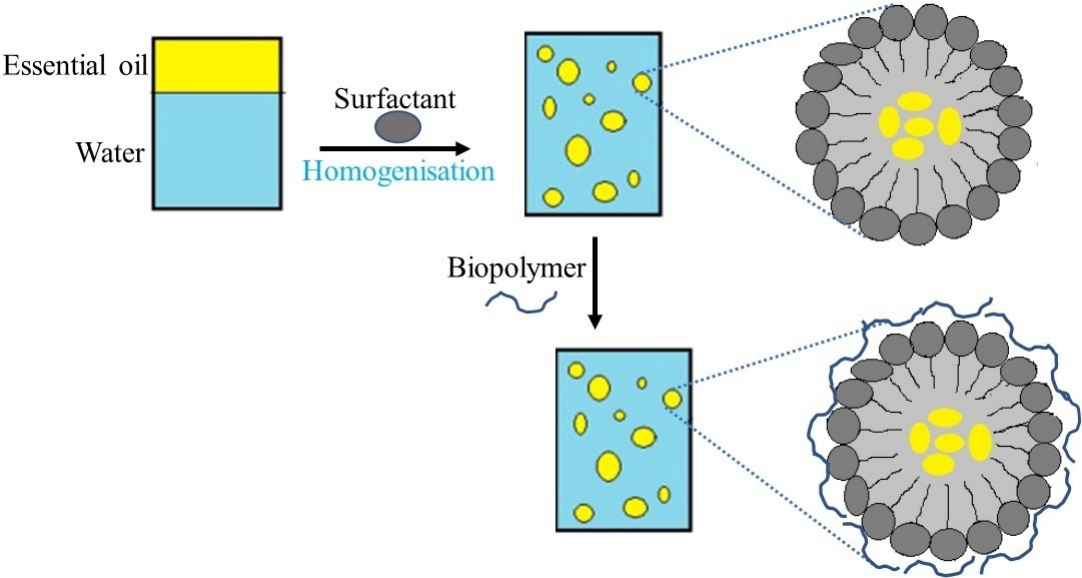
Nanoencapsulation of plant extract by emulsion.

Another process reported for the encapsulation of the oil extracted from *O. basilicum* is the lyophilization technique, also known as freeze‐drying. Treatment of a mixture of the essential oil, aroma (5 %), and acacia gum‐maltodextrin (45 %) in water (50 %) using a homogenizer, and then lyophilization of the frozen emulsions, produced particles with increased insecticidal activity. The findings indicated that >80 % mortality of pest *Rhyzopertha dominica* was recorded at 1 g/kg dose *via* contact and ingestion toxicity. Similar observations were also reported by Balasubramani and coworkers with the nanoemulsion droplets (<200 nm) of *O. basilicum* leaf essential oil (Sundararajan et al., 2018). These were generated using the emulsion of 5 % (w/v) essential oil in 90 % (w/v) water, in the presence of 5 % (w/v) hydrophilic surfactant Polysorbate 80. Emulsions in the nanometer‐scale are heterogeneous dispersions generated by stabilizing two immiscible liquids (oil and water) using small amounts (<10 %) of a surfactant,^[80]^ as shown in Figure 6.

**Figure 7:**
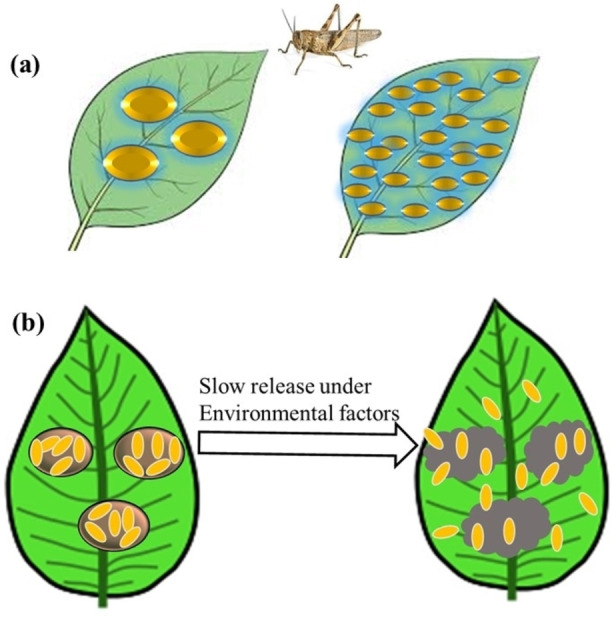
Representation of (a) pesticide distribution on leaf surface based on size, and (b) slow release of active ingredients from carriers.

An organic solvent‐free technology based on the mass transfer properties of fluid under supercritical conditions has been described to yield nanoemulsions with relatively reduced size.^[84]^ Plant extracts have also been processed using this eco‐friendly method. As depicted in Figure 7, decreasing particle size results in good spatial distribution of the control agents on the surface of the leaves.

Mendonca et al.^[73]^ used the supercritical CO_2_ extraction process to develop stable nanoemulsions of *A. indica* seed oil and biodegradable poly(3‐hydroxybutyrate‐co‐3‐hydroxyvalerate), with smaller size of 228 nm in comparison with the initial emulsion droplets (463 nm). However, these sphere‐shaped nanoparticles had lower maximum EE (13 %). Aqueous nanoemulsions loaded with plant extracts increase the solubility of phytochemicals in water, thus avoiding the use of hazardous organic solvents.^[23]^

Nanoscale carriers not only contributes to preserving the long‐term viability of plant‐derived pesticides but can significantly improve the fate of their active ingredients through a timely release behavior. The controlled delivery of phytochemicals by nanoencapsulated systems is one strategy that can integrate safety, efficacy, and sustainable use of resources to preclude the desert locust infestation. Hitherto, several studies have probed the outcomes of botanical pesticide nanoencapsulation for reducing reliance on traditional synthetic pesticides and these are presented in Table 2.

**Table 2:** Nanoencapsulation of active pesticidal plant extracts using polymeric nanostructured carriers.

Plant extract (Formulation)	Carrier material	Method of encapsulation	References
*P. granatum* (hydro‐methanol peel extract)	Chitosan	Ionotropic gelation using TPP as a crosslinking agent. Particles AS: 198 nm EE: 26–70 %	^[99]^
N. oleander (methanol leaf extract)	Chitosan	Ionotropic gelation with TPP.	^[4]^
*P. granatum* (hydro‐ethanol fruit peel extract)	Alginate	Ionotropic gelation using Ca2+ as a crosslinker and span 80 as surfactant. AS: 205 nm EE: 57–84 %	^[92]^
*A. indica* (aqueous leaf extract)	Alginate	Ionotropic gelation using Ca2+ as a crosslinker.	^[95]^
*P. harmala* (ethanol seed extract)	p‐Sulfocalix[6]arene	Thin‐film hydration process at 40 °C using methanol. AS: 265 nm EE: 74–89 %	^[34]^
	Poly(lactic‐co‐glycolic acid) (PLGA)‐polyethylene glycol (PEG)	Multiple emulsion‐solvent evaporation method AS: 208 nm EE: 81–87 %	^[36]^
*P. harmala* (ethanol seed extract)	Chitosan‐PLGA	Emulsification‐solvent evaporation using polyvinyl alcohol as surfactant. AS: 202 nm EE: 25–88 %	^[16]^
*P. harmala* (ethanol leaf and fruit extract)	Chitosan	Emulsification‐ionotropic gelation with TPP. Ultra‐sonication AS: 210 nm EE: 80–87 %	^[53]^
*A. indica* (seed oil)	Poly(3‐hydroxybutyrate‐co‐3‐hydroxyvalerate)	Emulsions stabilized in the presence of poly (vinyl alcohol) under stirring at 40 °C. Supercritical fluid extraction using CO2. AS: 153–307 nm EE: 9–13 %	^[73]^
*M. chamomilla* (essential oil)	Surfactin‐tween 80	Emulsification AS: 341 nm	^[50]^
*C. cyminum* (essential oil)	Surfactin‐tween 80	emulsification AS: 387 nm	
*M. chamomilla* (essential oil)	Tween 80	Emulsification AS: 130 nm	^[1]^
*O. basilicum* (essential oil)	Tween 80	Emulsification AS: 129 nm	
*C. cyminum* (essential oil)	Tween 80	Emulsification AS: 173 nm	
*O. basilicum* (leaf oil)	Maltodextrins‐acacia gum	Freeze‐drying	^[27]^
*O. basilicum* (leaf oil)	Polysorbate 80	Emulsification AS: 200 nm	^[19]^
*O. basilicum* (essential oil)	Chitosan‐tween 80	Emulsification‐ionotropic gelation with TPP. AS: 199–373 nm EE: 5–50 %	^[25]^
*C. cyminum* (seed oil)	Chitosan‐myristic acid	Self‐assembly technique using 1‐ethyl‐3‐(3‐dimethylaminopropyl)‐carbodiimide as crosslinker. Ultra‐sonication in water bath. AS: 30–250 nm	^[113]^
*L. sativum* (essential oil)	Soybean lecithin‐tween 80	Ultrasonic‐solvent emulsification technique.	^[6]^
*N. sativa* (edible oil)	Tween 80‐sodium dodecyl sulfate‐cetyltrimethylammonium bromide	Ultrasonic‐solvent emulsification with ethanol as co‐surfactant. AS: 100–224 nm	^[10]^
*O. vulgare* (essential oil)	Lipoid S100 Phospholipon 85 G Phospholipon 90H	Ethanol injection technique using cholesterol as surfactant. AS: 81–319 nm EE: 83–85 %	^[65]^

## 5 Development of Phytochemical‐Based Metal Nanopesticides

**Figure 8:**
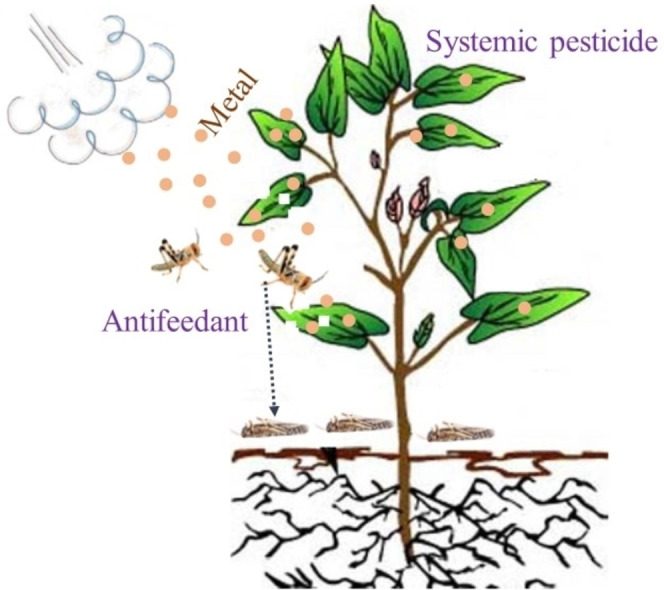
Metal contribution to pesticidal activity.

The hyperaccumulation of metal ions in plants has been documented to provide a unique chemical defense against insect pests (Figure 8).[^22^, ^42^, ^72^] The production of secondary metabolites that have toxic or repellent effects on insect pests can be enhanced by metal ions in various plant species.[^39^, ^82^] These are phytochemicals that have no essential role in the growth and development of plants but play significant characters on plant‐environment interactions: adaptation to abiotic factors and defense to biotic stress. A study conducted by Behmer et al.,^[22]^[Q1] [Q2]  on the feeding behavior of *S. gregaria* on *Thlaspi caerulescens* species, revealed predilection for low Zn‐concentrated plants. The results indicated that plants containing a relatively low mean Zn concentration of 415 μg g^−1^ were preferred by fourth instar nymphs, as opposed to intermediate (1502 μg g^−1^) and high (5774 μg g^−1^) Zn concentrated analogues. A recent study by Hashem et al.^[51]^ demonstrates that the 26 nm zinc chromium oxide can trigger structural damages in the fat body cells of fifth instar nymphs. Following injection of a Fe‐rich solution, a significant increase in antioxidant enzyme activities and oxidative tissue damage in the gut of immature stages were also reported due to disproportionate amounts of reactive oxygen species (ROS) (Renault et al., 2016). Moreover, deposition of metals at the surface of plants has been argued to cause detrimental effects on feeding insects.^[89]^ A previous investigation on the foliar application of copper‐containing nanoparticles (Cu(OH)_2_ 16 nm, CuO 38 nm, and Cu(OH)_2_ 50 nm) has established that metal‐based nanoparticles adhere to the plant surface for many days even after washing.^[66]^ The size of these metal‐based nanoparticles is also likely to influence their ability to clog in the apoplast. The pores within cellulose/hemicellulose cell walls have been found to range between 5 to 20 nm.^[15]^ These findings suggest superior and long‐term efficacy of metal nanoparticles in the management of *S. gregaria* as compared with their ionic counterparts.

Metal nanoparticles can oxidize in biological media to generate metal ions as oxidative stressors in insects. Apart from this, the nanometer‐scale has been found to assist their penetration through larvae membranes and trigger the oxidative stress‐mediated signaling pathways that contribute to tissue injury. Also, the pesticidal activity of metal nanoparticles has been associated with the alteration/leakage of cellular membrane and the inhibition of respiratory activity.^[7]^ Notably, surface capping with phytochemicals can magnify these actions by providing paramount stability, enhancing reactivity, and improving dispersion in aqueous media.[^31^, ^58^] Further, the high surface to volume ratio of metal nanoparticles enables high adsorption of phytochemicals and eventually increases their bioactivities.^[78]^ In view of the adhesion of metal nanoparticles to crop foliage, attaching the active ingredients onto their surface reduces pesticide run‐off.

**Figure 9:**
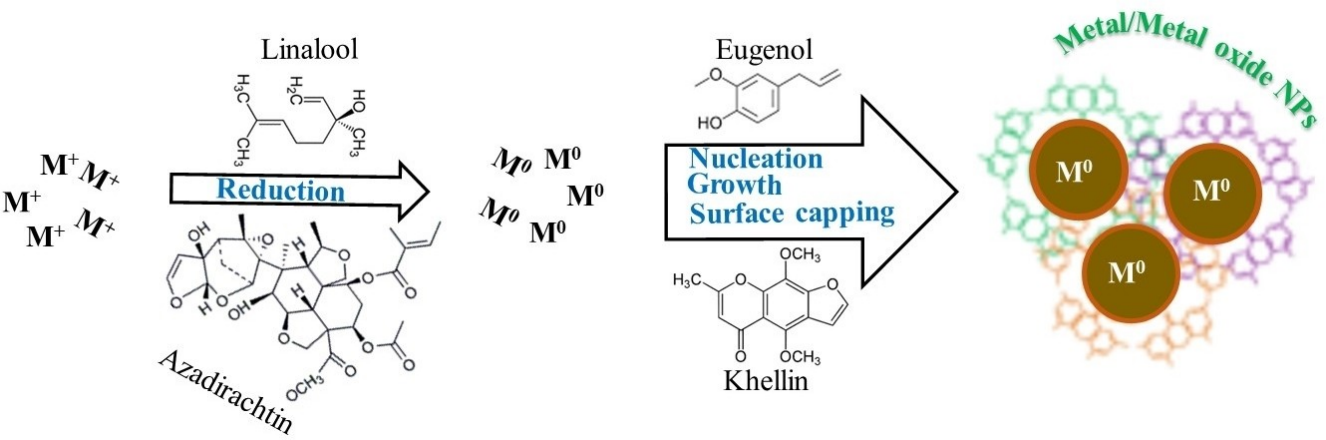
Schematic illustration of the phytochemical‐mediated formation of metal/metal oxide nanoparticles.

The surface coating of metal nanoparticles with bioorganic compounds occurs during the reduction of metal ions in the presence of hydroxyl‐rich phytochemicals that serve as electron donors.^[90]^ It can also be postulated that plant extracts contain compounds with nucleophilic (−C(−O(−, (−C(=O, (−C(−O(−C(−, and (−C(=C(−) functional groups, which can facilitate the conversion of metal ions to nanoscale zero‐valent metal. Subsequently, various interactions, such as metal coordination, hydrogen bonding, electrostatic attraction, and π–π stacking transpire to assemble the phytochemicals at the surface of the growing metal nanoparticles,^[111]^ The interaction between the surface of metal nanoparticles and the chains of bioorganic ligands leads to steric hindrance, thus preventing agglomeration (Figure 9). Metal ions like Ag^+^, Fe^2+^, Fe^3+^, Pt^4+^, and Pd^2+^ interacting with the bioactive ingredients present in extracts from pesticidal plants have also been reported to produce nanoparticles with different sizes and pesticidal characteristics, as shown in Table 3.

**Table 3:** Preparation of phytochemical‐coated metal nanoparticles with pesticidal activity.

Plant species (Formulation)	Metal‐containing nanoparticles (NPs)	Method of preparation	Representative insect pests	Significant insecticidal results	References
*A. indica* (leaf extract)	Spherical Ag NPs AS: 7–10 nm	Stirred mixture of an aqueous solution of AgNO_3_ and plant extract at 85 °C for 30 min.	*Bemisia tabaci*	‐LC_50_: 411.054 and 465.331 ppm, against 3rd instar nymphs and adult population of whitefy, respectively, after 96 h exposure time.	^[98]^
*A. indica* (neem gum suspension)	Rutile TiO_2_ NPs Size: 20–41 nm)	Autoclave treatment of an aqueous suspension of neem gum and TiCl_4_.	*Spodoptera litura* and *Helicoverpa armigera*	‐LC_50_: 10.20 and 12.49 ppm on *Helicoverpa armigera* (Hub.) and *Spodoptera litura* (Fab.), respectively; ‐LC_90_: 32.68 and 36.68 ppm on *H. armigera* (Hub.) and *S. litura* (Fab.), respectively.	Kamaraj et al., 2017
*P. harmala* (methanol seed extract)	Ag NPs AS: Size: 22–66 nm	Stirred mixture of an aqueous solution of AgNO_3_ and plant extract at 80 °C for 30–45 min. Incubation in the dark at 37 °C.	*Trogoderma granarium*	‐LC_50_ range (after 48 h post‐treatment): ‐In instar larvae of *Trogoderma granarium*: 4.7–11.4 μg/cm^2^; ‐In adults of *Trogoderma granarium*: 4.1–10.2 μg/cm^2^.	^[11]^
*P. harmala* (aqueous extract)	Spherical Ag NPs	Stirred mixture of an aqueous extract and AgNO_3_ solution at 60–70 °C for 1 h.	*Culex pipiens*	Killing rate of C. pipiens by the silver nanoparticle: ‐After 48 h of exposure: 3.3, 4.6, 4.6, 5.3 % at the concentrations of 2.5, 5, 7.5, and 10, respectively, vs untreated control (0); ‐After 24 h of exposure: 3.3, 3.0, 2.6, 4.6 % at the concentrations of 2.5, 5, 7.5, and 10, respectively, vs untreated control (0).	^[108]^
*P. harmala* (ethanol seed extract)	Spherical Pt−Pd NPs AS: 34 nm	Stirred mixture of seed extract and an aqueous solution of PtCl_4_ and Pd(OAc)_2_ at 60 °C for 24 h.	Not reported	IC_50_ values from cytotoxicity tests: 8.8 and 3.6 μg/ml, against lung cancer (A549) and breast adenocarcinoma (MCF‐7) cells, respectively; vs carboplatin (IC_50_: 23 and 9.5 μg/ml, respectively).	^[35]^
*O. basilicum* (aqueous leaf extract)	Spherical and octagonal Ag NPs AS: 14–36 nm	Stirred mixture of leaf extract and an aqueous solution of AgNO_3_ at ambient temperature for 1 h in the dark.	*Spodoptera litura*	Mortality rate of the nanoparticle in second instar larvae: 21.67–96.67 % at concentrations ranging from 100 to 1500 mg/l, vs coragen (18.33–91.67 %), proclaim (13.33–78.33 %), talstar (13.33–68.33 %), and tracer (11.6–66.67 %) at concentrations ranging of 60 to 120 mg/l.	^[56]^
*C. cyminum* (methanol seed extract)	Fe_3_O_4_ NPs AS: 20 nm	Stirred mixture of seed extract and an aqueous solution of FeCl_2_.4H_2_O FeCl_3_.6H_2_O at 50 °C for 1 h. pH Adjustment >10 Continuous stirring at 50 °C for 20 min.	*Lepidoptera gelechiidae*	LC_50_ values: ‐Fe_3_O_4_ NPs: 601.48, 268.82, and 595.16 ppm for penetrating neonate larvae to tubers and leaves, and eggs of *Phthorimaea operculella*, respectively, vs cumin extract (LC_50_: 961.07, 496.84, and 874.90 ppm, respectively).	^[62]^
*D. stramonium* (ethanol extract)	Ag NPs	Stirred mixture of extract and a solution of AgNO_3_ at 100 °C for 5 min.	*Culex pipiens*	‐LC_50_ values of the nanoparticles against the 3rd instar larvae of *Culex pipiens*: 112.92, 105.12, 95.91, and 84.11 ppm after 24, 48, 72 and 96 h of exposure; vs crude ethanol extract (LC_50_: 612.07, 1966.29, 520.63 and 455.45 ppm, respectively).	^[54]^
*D. stramonium* (leaf oil)	Ag NPs	Stirred mixture of oil extract and an aqueous solution of AgNO_3_ at ambient temperature for few min.	*Trogoderma granarium*	Percentage of mortality of the nanoparticle: 41.40 % at 300 ppm, vs plant oil (36.12 % of mortality at 15 % concentration).	^[104]^
*N. oleander* (aqueous leaf extract)	Spherical and cubic Ag NPs AS: 20–35 nm	Stirred mixture of leaf extract and an aqueous solution of AgNO_3_ under at ambient temperature. Incubation at room temperature.	*Anopheles stephensi*	LC_50_ values of the nanoparticle: ‐Against larvae: 20.60, 24.90, 28.22 and 33.99 ppm for first to fourth instar larvae, respectively; vs leaf aqueous extract (LC_50_: 232.90, 273.71, 318.94, and 369.96 ppm, respectively); ‐Against pupae: LC_50_: 426.01 ppm, vs leaf aqueous extract (426.01 ppm).	^[93]^
*N. oleander* (aqueous leaf extract)	Spherical shaped Ag NPs AS: 52 nm	Stirred mixture of leaf extract and an aqueous solution of AgNO_3_ in the dark at ambient temperature.	*Tribolium castaneum* and *Callosobruchus maculatus*	*Against *Callosobruchus maculatus*: LC_50_ value: 2.992 ppm for the green synthesized nanoparticle, vs aqueous leaf extract (LC_50_: 3.366 ppm), and pure nanoparticle (LC_50_: 4.332 ppm); *Against *Tribolium castaneum* larvae (first instar): LC_50_ value: 2.227 ppm, vs aqueous leaf extract (LC_50_: 3.853 ppm), and pure nanoparticle (LC_50_: 2.019 ppm).	^[55]^
*N. oleander* (methanol leaf extract)	Spherical formed Ag NPs AS: 4–32 nm	Stirred mixture of leaf extract and AgNO_3_ solution under darkness at room temperature.	*Lucilia sericata*	LC_50_ of the prepared nanoparticle against the third instar larvae of *L. sericata*: 21 ppm, vs *N. oleander* leaf methanol extract (LC_50_: 389 ppm).	^[3]^

## 6 Critical Assessment

The present study aims to summarize up‐to‐date information on the use of nanoformulations from pesticidal plant natural products against desert locust swarms. Indeed, detailed information regarding mode of preparation of nanoparticles from a series of plant species, materials and techniques of preparation employed, and their insecticidal and/or pesticidal activity are discussed.

According to the literature information, twenty‐two (22) plant species (Table 1) were reported to biologically affect the desert locust by impairment of hormonal regulation of metamorphosis, reduction of the fecundity of female species, increase in acetylcholinesterase enzymatic activity, a slowdown of locomotion, antifeeding activity, and so on. The plant organs that were mostly used for plant extraction included leaves, seeds, fruits, whole plant, etc. The main secondary metabolites, which were intricately involved in the biological activity included triterpenoids (β‐terpinene‐4‐ol, β‐terpinene, and sabinene‐phelandrene from Origanum vulgare), tropane alkaloids (scopolamine and hyoscyamine from Datura stramonium), furanochromones (khellin, visnagin and from Ammi visnaga), limonoid (azadirachtin) and flavonoids (quercetin and kaempferol from Nerium oleander) among others.

Despite the effectiveness of these plants against desert locusts, the toxicity caused by their overuse is noteworthy. For example, the ingestion of a significant amount of N. oleander leaf extract may cause serious health complications to children due to high concentrations of cardiac glycosides.^[20]^ Thus, detailed toxicity studies of the most promising plants should be investigated for their successful utilization in crop protection.

So far, encapsulation of plant extracts with promising insecticidal activity has been accomplished successfully in nanostructured carriers, to fulfil nano‐formulations with controlled release of pesticidal substances (Table 2). However, there is a lack of studies across the literature regarding assessment of the potential interaction of the systems carrier‐phytochemicals in insecticidal activity. Based on their biophysical properties, liposomal and biopolymeric nanocarriers can ease the permeation of encapsulated phytochemicals via the larval rectal epithelium of desert locusts, such as S. gregaria. Positively charged materials can interact with the epithelial cells’ surface, leading to cuticle damage and transmucosal delivery of active ingredients.[^67^, ^87^] Different mechanisms might expound the effect of nano‐enabled pesticides and these are well discussed in represented in the literature.[^57^, ^79^]

From the results displayed in Table 3, there is evidence that green synthesized nanoparticles exhibit more potent insecticidal activity than pure metals and plant extracts used as starting points for the fabrication. Rutile TiO_2_ NPs, Ag NPs, Pt−Pd NPs and Fe_3_O_4_ NPs were the mostly fabricated insecticidal nanoparticles with sizes varying from 4 to 66 nm. Despite the environmentally benign nature of the bioactive ingredients‐mediated preparation of metal nanopesticides, assessment of the environmental risk associated with metal nanoparticles for regulatory purposes continues to be a challenge. The conventional methods applied to monitor the residual nanoparticles in the environment are solely based on sequential extraction of dissolved concentrations and speciation.[^64^, ^103^] Considering the relatively small size (≤50 nm; Table 3) of the reported metal‐containing nanopesticides, more basic research should also be conducted on their ability to translocate within the plant. The release of pesticidal phytochemical‐coated metal nanoparticles in the environment even at lower concentration may result in long‐term higher toxicity on biota than the pristine active ingredient formulations or nanoencapsulated phytochemical pesticides.

## 7 Conclusions and Future Outlook

This study examines the current state of knowledge on the use of pesticidal plants and their nanoformulations for the control of swarming desert locusts. Over the past decade, modern biological studies on the insecticidal activity of essential oils and extracts from a collection of 17 plant families have been published. Studies of the insecticidal mechanism of action of these plant natural products revealed their implication in epithelial cell damage within the midgut of desert locusts. Other insecticidal mechanisms of action included interference of plant extracts with the central nervous system, and an increase in acetylcholinesterase enzymatic activity in the insect, and so forth. To prevent degradation of plant extracts under environmental stress, and preserve their systemic efficacy, encapsulation within nanostructures has been described.

Based on the findings of studies presented in this review, the following key insights emerged:

– The higher encapsulation efficiency of biopolymeric nanocarriers make them commendable delivery systems to maintain the safety, stability and efficacy of plant extracts used as biopesticides. The key fabrication techniques of these biopolymeric nanocarriers should focus on the limited use of hazardous solvents and the optimisation of solvent evaporation methods and lyophilisation to maintain minimal concentration of solvents in the final formulation.
– Capping silver nanoparticles with the bioactive compounds contained in plant extracts is anticipated to reduce pesticide run‐off and underpin a system with synergistic effects against desert locust. However, metal‐based nanopesticides if used, should only be considered at concentrations that are less toxic to the plant and where no harmful effects can occur to humans.
– Nanostructured biopesticides obtained using green technology such as super critical fluid are ideal as they eliminate the use of any organic solvents and are produced at optimal sizes, even smaller than biopolymeric nanoparticulate. However, the low encapsulation efficiency combined with the cost of this sophisticated technique makes it unattractive for emerging countries who attribute a sizeable percentage of their economy to agricultural activities.
– Nanotechnology offers the potential to develop more environmentally friendly pesticides. By improving targeting and reducing off‐target effects, nanoparticles can minimize the amount of pesticide needed, thus reducing pollution and the risk to non‐target organisms. Additionally, nanopesticides can be designed using biodegradable polymers to degrade more rapidly and safely in the environment.
– Nanotechnology enables the development of smart pesticides with increased precision and specificity that can specifically target pests while sparing beneficial organisms. By functionalizing nanoparticles with targeting ligands, such as antibodies or peptides, nanopesticides can selectively bind to pests or their specific receptors, reducing the impact on non‐target organisms.
– Challenges may including scaling up the production of nanopesticides to meet agricultural demands. The cost of manufacturing nanoparticles and incorporating them into pesticide formulations would need to be competitive with existing conventional pesticides.
– Furthermore, the potential toxicity of the use of nanoparticles in developing based plant pesticides and their effects on human health and the environment is a significant concern. Therefore, rigorous research is needed to understand the potential risks associated with the use of such nanopesticides and to develop appropriate safety regulations.

In conclusion it can be said that the in‐depth understanding provided on the development processes, performance and interaction of nanotechnology‐based plant pesticides with crops, will open new insights in the production of more effective plant‐based biopesticides as a sustainable alternative against desert locusts.

## Copyright Statements

All the figures/graphics used in this review article were created originally.

## Conflict of Interests

The authors declare no conflict of interest.

8

## Biographical Information

*Dr. Hugues Kamdem Paumo is a research associate at North‐West University. He obtained his MSc (2014) and PhD (2017) in Chemistry at the University of South Africa under the supervision of Prof. MJ Mphahlele. He then joined the water research group of Prof. Arjun Maity at CSIR, South Africa from 2017 to 2019. He served as postdoctoral research fellow at North‐West University (2020 to 2023) under the supervision of Prof. LM Katata‐Seru. He is interested in the development of nanomaterials with potential application in water reclamation, pharmaceutical, and agriculture*.

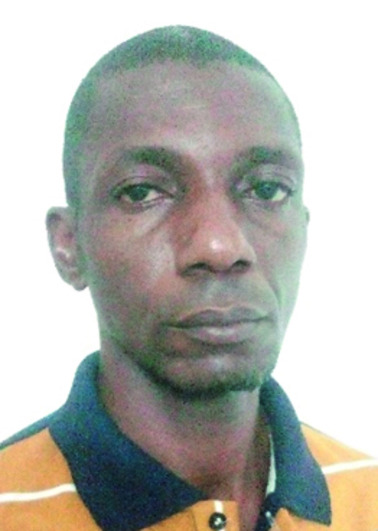

## Biographical Information

*Prof Lebogang Katata‐Seru works at the North‐West University in South Africa (Mafikeng Campus) as an Associate Professor (Chemistry), Director (School of Physical and Chemical Sciences) and Acting Deputy Dean (Community Engagement and Stakeholder Relations). She has published research work in peer‐reviewed journals and book chapters. To date, she has managed to supervise several M & D students and postdoctoral fellows to completion in the field of nanotechnology. She has reviewed various M&D dissertations and theses from universities, such as international manuscripts from Pharmaceutical Nanotechnology, Journal of Nanoparticle Research, etc. Furthermore, she was the Winner of the Public Enterprises & Institutions Sector for SA MIW 2012. She is also a member of various professional bodies i. e., Royal Society of Chemistry, British Society for Nanomedicine, IUPAC, etc. Her research focus is nanoformulating compounds for possible applications in various agricultural fields*.

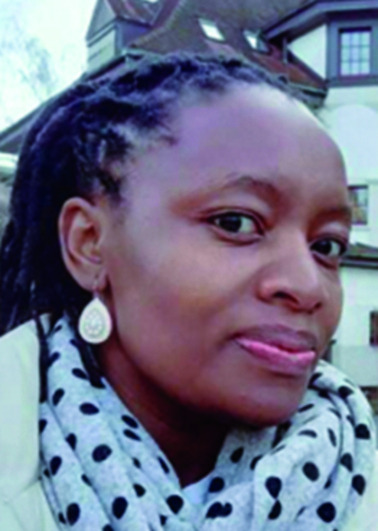

## Data Availability

The data that support the findings of this study are available from the corresponding author upon reasonable request.

## References

[1] K. Abdelaal , M. Essawy , A. Quraytam , F. Abdallah , H. Mostafa , K. Shoueir , Y. Hafez , Processes 2021, 9(4), 1–14.

[2] Z. A. S. Abdelatti , M. Hartbauer , J. Pest Sci. 2020, 93(1), 341–353.

[3] A. D. Abdel-Meguid , Int. J. Trop. Insect Sci. 2022, 42(3), 2579–2591.

[4] A. Abdel-Meguid , R. H. Ramadan , M. Emara , Catrina: The Int. J. Environ. Sci. 2020, 21(1), 9–14.

[5] A. O. Abdelbagi , M. N. E. H. E. Amin , A. E. S. A. Ishag , A. M. A. Hammad , Afr. J. Agric. Res. 2019, 14(32), 1472–1486.

[6] S. M. Abd-El Wahed , D. A. Youssef , M. M. Adel , Plant Arch. 2021, 21(1), 770–778.

[7] B. Ahmed , F. Ameen , A. Rizvi , K. Ali , H. Sonbol , A. Zaidi , M. S. Khan , J. Musarrat , Am. Chem. Soc. Omega 2020, 5(14), 7861–7876. 10.1021/acsomega.9b04084 PMC7160826 32309695

[8] A. Aoudia , A. Kemassi , A. Herouini , A. Taibaoui , Z. Derouiche , R. Bouziane , N. Cherif , R , M. D. Ould el Hadj , Acta Entomol. Serbica 2021, 26(2), 1–17.

[9] A. Aoudia , A. Kemassi , A. Herouini , Z. Taibaoui , R. Derouiche , N. Bouziane , R. Cherif , M. D. Ould el Hadj , Acta Zoológica Lilloana 2022, 66(2), 161–178.

[10] S. A. Ali , M. Khairy , A. A. Ibrahim , N. M. Zohry , J. Food Sci. 2022, 87, 3095–3106. 35638325 10.1111/1750-3841.16186

[11] A. A. Almadiy , G. E. Nenaah , D. M. Shawer , J. Pest Sci. 2018, 91(2), 727–737.

[12] Z. I. A. Al-Fifi , J. Entomol. 2006, 3(4), 312–318.

[13] N. A. Al-Maroug , M. I. Nassar , G. M. Abdelatef , M. M. ELShazly , E. A. E. A. ElFattah , D. H. A. El-Monem , Egypt. Acad. J. Biol. Sci. F. Toxicol. Pest Control 2022, 14(2), 121–132.

[14] M. A. M. Al Sulaibi , C. Thiemann , T. Thiemann , Open Chem. J. 2020, 7(1), 1–15.

[15] A. Avellan , J. Yun , B. P. Morais , E. T. Clement , S. M. Rodrigues , G. V. Lowry , Environ. Sci. Technol. 2021, 55(20), 13417–13431. 33988374 10.1021/acs.est.1c00178

[16] H. M. E. S. Azzazy , S. A. Fahmy , N. K. Mahdy , M. R. Meselhy , U. Bakowsky , Nanomaterials (Basel) 2021, 11(9), 1–16. 10.3390/nano11092438 PMC8464825 34578755

[17] L. Badisco , J. Huybrechts , G. Simonet , H. Verlinden , E. Marchal , R. Huybrechts , L. Schoofs , A. De Loof , J. Vanden Broek , PLoS One 2011, 6, 1–22. 10.1371/journal.pone.0017274 PMC3061863 21445293

[18] M. Bagari , A. Bouhaimi , S. Ghaout , J. Chihrane , Zoologica Baetica 2013, 24(1), 193–203.

[19] S. Balasubramani , A. K. Moola , K. Vivek , B. R. Kumari , Microb. Pathogen. 2018, 125, 475–485. 30340015 10.1016/j.micpath.2018.10.017

[20] V. Bandara , S. A. Weinstein , J. White , M. Eddleston , Toxicon 2010, 56(3), 273–281. 20438743 10.1016/j.toxicon.2010.03.026

[21] E. M. Bashir , H. A. El Shafie , Am. J. Exp. Agric. 2014, 4(8), 959–970.

[22] S. T. Behmer , C. M. Lloyd , D. Raubenheimer , J. Stewart-Clark , J. Knight , R. S. Leighton , F. A. Harper , J. A. C. Smith , Funct. Ecol. 2005, 19(1), 55–66.

[23] E. B. Buzanello , G. T. B. P. Machado , S. Kuhnen , L. Mazzarino , M. Maraschin , Nano Exp. 2020, 1(1), 010058.

[24] N. R. Cabej, *Epigenetic Principles of Evolution (Second Edition)*, **2019**.

[25] M. Cai , Y. Wang , R. Wang , M. Li , W. Zhang , J. Yu , R. Hua , Int. J. Biol. Macromol. 2022, 202, 122–129. 35041880 10.1016/j.ijbiomac.2022.01.066

[26] M. P. Chapuis , A. Foucart , C. Plantamp , L. Blondin , N. Leménager , L. Benoit , P. E. Gay , C. S. Bazelet , Afr. Entomol. 2017, 25(1), 13–23.

[27] M. Chenni , D. El Abed , S. Neggaz , N. Rakotomanomana , X. Fernandez , F. Chemat , J. Stored , Prod. Res. 2020, 86, 101575.

[28] B. Çiplak , Agronomy 2021, 11(1), 111.

[29] D. A. Cullen , G. A. Sword , T. Dodgson , S. J. Simpson , J. Insect Physiol. 2010, 56, 937–942. 20438734 10.1016/j.jinsphys.2010.04.023

[30] B. Dhlamini , H. K. Paumo , B. P. Kamdem , L. Katata-Seru , I. Bahadur , J. Environ. Chem. Eng. 2022, 10(3), 107729.

[31] A. M. El Shafey , Green Process. Synth. 2020, 9(1), 304–339.

[32] N. E. El-Wakeil , Gesunde Pflanzen 2013, 65(4), 125–149.

[33] U. R. Ernst , M. B. Van Hiel , G. Depuydt , B. Boerjan , A. De Loof , L. Schoofs , J. Exp. Biol. 2015, 218(Pt 1), 88–99. 25568455 10.1242/jeb.107078

[34] S. A. Fahmy , M. Y. Issa , B. M. Saleh , M. R. Meselhy , H. M. E. S. Azzazy , Am. Chem. Soc. Omega 2021 a 6(18), 11954–11963. 10.1021/acsomega.1c00455 PMC8153973 34056350

[35] S. A. Fahmy , I. M. Fawzy , B. M. Saleh , M. Y. Issa , U. Bakowsky , H. M. E. S. Azzazy , Nanomaterials (Basel) 2021 b, 11(4), 965. 33918743 10.3390/nano11040965 PMC8103518

[36] S. A. Fahmy , N. K. Mahdy , H. Al Mulla , A. N. ElMeshad , M. Y. Issa , H. M. E. S. Azzazy , Pharmaceutics 2022, 14(1), 142. 35057040 10.3390/pharmaceutics14010142 PMC8780513

[37] T. Farkhondeh , M. Kianmehr , T. Kazemi , S. Samarghandian , M. R. Khazdair , Hum. Exp. Toxicol. 2020, 39(6), 773–784. 31971021 10.1177/0960327120901571

[38] Z. Fathy , J. Muhammad , A. Azazy , J. Invertebr. Pathol. 2023, 198, 107922. 37068730 10.1016/j.jip.2023.107922

[39] M. A. Fazili , I. Bashir , M. Ahmad , U. Yaqoob , S. N. Geelani , Bull. Natl. Res. Centre 2022, 46(1), 35. 10.1186/s42269-022-00717-z PMC8857880 35221660

[40] M. J. G. Fernandes , R. B. Pereira , A. R. O. Rodrigues , T. F. Vieira , A. G. Fortes , D. M. Pereira , S. F. M. Sousa , S. T. Gonçalves , E. M. S. Castanheira , Nanomaterials (Basel) 2022, 12(20), 1–17. 10.3390/nano12203583 PMC9611868 36296773

[41] F. A. O., *Greater Horn of Africa and Yemen–Desert locust crisis appeal*, January–December, Rapid response and sustained action, revised edition. Rome, **2020**.

[42] J. E. Gall , R. S. Boyd , N. Rajakaruna , Environ. Monit. Assess. 2015, 187(4), 1–21. 25800370 10.1007/s10661-015-4436-3

[43] B. García-Pinel , C. Porras-Alcalá , A. Ortega-Rodríguez , F. Sarabia , J. Prados , C. Melguizo , J. M. López-Romero , Nanomaterials (Basel) 2019, 9(4), 638. 31010180 10.3390/nano9040638 PMC6523119

[44] K. Ghoneim , M. Amer , A. Al-Daly , A. Mohammad , F. Khadrawy , M. Mahmoud , Int. J. Biol. Sci. 2014 a, 5(1), 397–414.

[45] K. Ghoneim , M. Amer , A. Al-Daly , A. Mohammad , F. Khadrawy , M. A. Mahmoud , Entomol. Appl. Sci. Lett. 2014 b, 1(2), 9–19.

[46] K. E. Ghoneim , K. S. Hamadah , A. A. El-Hela , J. Entomol. Res. Soc. 2013, 14(2), 87–97.

[47] K. Ghoneim , K. Hamadah , A. El-Hela , J. Biol. Res. (Thessalon) 2016, 1(2), 1–10.

[48] J. Guan , M. Li , X. Ju , J. Lin , J. Wu , J. Zheng , Peer J. 2021, 9, 1–25. 10.7717/peerj.12311 PMC8555501 34754618

[49] D. Haouas , In Agriculture Productivity in Tunisia Under Stressed Environment. Springer Cham, 2021, 137–154.

[50] A. S. Hashem , M. M. Ramadan , J. Plant Protect. Pathol. 2021, 12(1), 11–17.

[51] F. M. Hashem , E. Elgazzar , W. A. Mostafa , BMC Chem. 2023, 17(1), 7. 36803540 10.1186/s13065-023-00914-5 PMC9940394

[52] A. A. Hatamleh , M. Danish , M. A. Al-Dosary , M. El-Zaidy , S. Ali , Royal Soc. Chem. Adv. 2022, 12(12), 7237–7252. 10.1039/d1ra09440h PMC8982233 35424659

[53] M. Izadi , S. A. M. Jorf , M. Nikkhah , S. Moradi , Physiol. Mol. Plant Pathol. 2021, 116, 1–7.

[54] A. Jabbar , M. Tariq , A. Gulzar , T. Mukhtar , T. Zainab , J. Zool. 2021, 54, 1259–1267.

[55] F. S. Jafer , M. R. Annon , J. Global Pharm. Technol. 2018, 10(3), 448–454.

[56] M. Jafir , J. N. Ahmad , M. J. Arif , S. Ali , S. J. N. Ahmad , Ecotoxicol. Environ. Safety 2021, 218, 1–9. 10.1016/j.ecoenv.2021.112278 33965777

[57] M. Jafir , M. Irfan , M. Zia-ur-Rehman , F. Hafeez , J. N. Ahmad , M. A. Sabir , U. Zulfiqar , R. Iqbal , F. Zulfiqar , A. Moosa , Plant Stress 2023, 10, 100208.

[58] R. Javed , M. Zia , S. Naz , S. O. Aisida , Q. Ao , J Nanobiotechnol. 2020, 18(1), 1–15. 10.1186/s12951-020-00704-4 PMC7682049 33225973

[59] N. Kaidi , C. Amroun , D. Hocine , S. E. Doumandji , D. Ghezali , Adv. Environ. Biol. 2017, 11(4), 37–45.

[60] C. Kamaraj , P. R. Gandhi , G. Elango , S. Karthi , I. M. Chung , G. Rajakumar , Int. J. Biol. Macromol. 2018, 107, 59–69. 28860055 10.1016/j.ijbiomac.2017.08.145

[61] A. Kemassi , N. Bouziane , Z. Boual , M. D Ould El Hadj , Phytothérapie 2014, 12(6), 348–353.

[62] F. Khorrami , K. Ojaghi Aghbash , A. Soleymanzade , M. Forouzan , Y. Ghosta , Acta Phytopathologica et Entomologica Hungarica 2019, 54(2), 243–251.

[63] J. N. Kinyuru , Int. J. Trop. Insect Sci. 2021, 41(3), 1993–1999.

[64] R. S. Kookana , A. B. A. Boxall , P. T. Reeves , R. Ashauer , S. Beulke , Q. Chaudhry , G. Cornelis , T. F. Fernandes , J. Gan , M. Kah , I. Lynch , J. Ranville , C. Sinclair , D. Spurgeon , K. Tiede , P. J. Van den Brink , J. Agric. Food. Chem. 2014, 62(19), 4227–4240. 24754346 10.1021/jf500232f

[65] T. L. Kryeziu , E. Haloci , A. Loshaj-Shala , U. Bagci , A. Oral , G. J. Stefkov , A. Zimmer , M. Basholli-Salihu , Die Pharmazie-An Int. J. Pharm. Sci. 2022, 77(6), 172–178. 10.1691/ph.2022.1230 35751165

[66] S. Laughton , A. Laycock , F. von der Kammer , T. Hofmann , E. A. Casman , S. M. Rodrigues , G. V. Lowry , J. Nanoparticle Res. 2019, 21(8), 1–13.

[67] J. Leal , H. D. Smyth , D. Ghosh , Int. J. Pharm. 2017, 532(1), 555–572. 28917986 10.1016/j.ijpharm.2017.09.018 PMC5744044

[68] G. M. W. Lengai , J. W. Muthomi , E. R. Mbega , Sci. Afr. 2020, 7, e00239.

[69] K. O. Maeno , S. O. Ely , S. A. O. Mohamed , M. E. H. M. E. H. Jaavar , M. A. O. B. Ebbe , Agronomy 2020, 10(12), 1–17.

[70] N. M. Mahdy , M. I. Mohammed , M. A. Abdou , S. S. Ahmed , N. S. Badawy , Pest Control 2019, 11(2), 37–58.

[71] S. A. Mansour , N. A. Abdel-Hamid , Ind. Crops Prod. 2015, 76, 900–909.

[72] S. N. Martens , R. S. Boyd , Oecologia 1994, 98(3), 379–384. 28313915 10.1007/BF00324227

[73] F. M. R. Mendonça , A. E. Polloni , A. Junges , R. S. da Silva , A. F. Rubira , G. R. Borges , C. Dariva , E. Franceschi , J. Supercrit. Fluids 2019, 152, 1–42.

[74] S. Messgo-Moumene , D. E. Merzouk , Z. Houmani , K. Moumene , Tunisian J. Plant Protect. 2015, 10(2), 117–130.

[75] J. S. Mossa , M. Tariq , A. Mohsin , A. M. Ageel , M. A. Al-Yahya , M. S. Al-Said , S. Rafatullah , Medicine (Baltimore) 1991, 19(3–4), 223–231. 10.1142/S0192415X91000302 1767794

[76] W. C. Mullié , A. Prakash , A. Müller , E. Lazutkaite , Agronomy 2023, 13, 819.

[77] M. I. Nassar , N. A. Ghazawy , H. M. Torkey , S. M. Rawy , Entomol. Appl. Sci. Lett. 2018, 5(2), 42–54.

[78] N. T. T. Nguyen , L. M. Nguyen , T. T. T. Nguyen , T. T. Nguyen , D. T. C. Nguyen , T. V. Tran , Environ. Chem. Lett. 2022, 20, 2531–2571. 35369682 10.1007/s10311-022-01425-w PMC8956152

[79] D. Nie , J. Li , O. Xie , L. Ai , C. Zhu , Y. Wu , Q. Gui , L. Zhang , W. Tan , Bioinorg. Chem. Appl. 2023, 5898160, 13. 10.1155/2023/5898160 PMC10195175 37213220

[80] M. D. Nuruzzaman , M. M. Rahman , Y. Liu , R. Naidu , J. Agric. Food. Chem. 2016, 64(7), 1447–1483. 26730488 10.1021/acs.jafc.5b05214

[81] S. A. Opiyo , Basic Sci. Med. 2020, 9(2), 32–37.

[82] S. Pagare , M. Bhatia , N. Tripathi , S. Pagare , Y. K. Bansal , Curr. Trends Biotechnol. Pharm. 2015, 9(3), 293–304.

[83] K. Pari , P. J. Rao , C. Devakumar , J. N. Rastogi , J. Nat. Prod. 1998, 61(1), 102–104. 9548837 10.1021/np970255z

[84] H. Park , J. S. Kim , S. Kim , E. S. Ha , M. S. Kim , S. J. Hwang , Pharmaceutics 2021, 13(11), 1928. 34834343 10.3390/pharmaceutics13111928 PMC8625501

[85] N. Pedrini , A. Ortiz-Urquiza , C. Huarte-Bonnet , S. Zhang , N. O. Keyhani , Front. Microbiol. 2013, 4, 1–18. 23422735 10.3389/fmicb.2013.00024 PMC3573267

[86] W. Peng , N. L. Ma , D. Zhang , Q. Zhou , X. Yue , S. C. Khoo , H. Yang , R. Guan , H. Chen , X. Zhang , Y. Wang , Z. Wei , C. Suo , Y. Peng , Y. Yang , S. S. Lam , C. Sonne , Environ. Res. 2020, 191, 1–7. 10.1016/j.envres.2020.110046 32841638

[87] H. Perumalsamy , J. R. Kim , S. M. Oh , J. W. Jung , Y. J. Ahn , H. W. Kwon , Public Lib. Sci. One 2013, 8(11), 1–9. 10.1371/journal.pone.0080226 PMC3832413 24260359

[88] D. Pimentel , J. Agri. Environ. Ethics 1995, 8(1), 17–29.

[89] C. Poschenrieder , R. Tolrà , J. Barceló , Trends Plant Sci. 2006, 11(6), 288–295. 16697693 10.1016/j.tplants.2006.04.007

[90] M. Pradeep , D. Kruszka , P. Kachlicki , D. Mondal , G. Franklin , Am. Chem. Soc. Sustain. Chem. Eng. 2021, 10(1), 562–571.

[91] R. E. Price , Insects 2023, 14, 846.37999045

[92] P. Rahnemoon , M. Sarabi-Jamab , A. Bostan , E. Mansouri , Food Biosci. 2021, 43, 1–8.

[93] M. Roni , K. Murugan , C. Panneerselvam , J. Subramaniam , J. S. Hwang , Parasitol. Res. 2013, 112(3), 981–990. 23239092 10.1007/s00436-012-3220-3

[94] A. A. M Salih , M. Baraibar , K. K. Mwangi , G. Artan , Nat. Clim. Change 2020, 10(7), 584–585.

[95] N. H. M. Salleh , N. A. Aziz , A. R. Mohamed , In American Institute of Physics Conference Proceedings. American Institute of Physics Publishing LLC 2021, 2347(1), 020143

[96] S. Sarker , Preprints 2022, 1, 2022060078.

[97] P. Satyal , S. Shrestha , W. N. Setzer , Nat. Prod. Commun. 2015, 10(8), 1453–1457. 26434140

[98] M. Shahid , U. Naeem-Ullah , W. S. Khan , S. Saeed , K. Razzaq , Int. J. Trop. Insect Sci. 2022, 42, 2443–2454.

[99] M. Soltanzadeh , S. H. Peighambardoust , B. Ghanbarzadeh , M. Mohammadi , J. M. Lorenzo , Nanomaterials (Basel) 2021, 11(6), 1–18. 10.3390/nano11061439 PMC8228277 34072520

[100] S. Srivastava , A. K. Srivastava , Appl. Biochem. Biotechnol. 2013, 171(6), 1351–1361. 23955295 10.1007/s12010-013-0432-7

[101] A. Tanani , S. Ghoneim , L. Basiouny , Egypt. Acad. J. Biol. Sci. 2009, 1(1), 45–55.

[102] C. Thiour-Mauprivez , F. Martin-Laurent , C. Calvayrac , L. Barthelmebs , Sci. Total Environ. 2019, 684, 314–325. 31153078 10.1016/j.scitotenv.2019.05.230

[103] F. Tou , Y. Yang , J. Feng , Z. Niu , H. Pan , Y. Qin , G. Xingpan , X. Meng , M. Liu , M. F. Hochella , Environ. Sci. Technol. 2017, 51(9), 4831–4840. 28380301 10.1021/acs.est.6b05931

[104] G. M. Umair , Z. Threem , S. Muhammad , I. M. Usama , I. Talal , GSC Biol. Pharm. Sci. 2020, 10(3), 010–015.

[105] A. Upadhyay , A. K. Rai , S. K. Verma , L. Upadhyay , A. Khan , O. Singh , S. Rout , Int. J. Environ. Clim. Change 2023, 13(7), 617–629.

[106] A. Van Huis , J. Insects Food Feed 2021, 7(3), 245–248.

[107] B. Wang , E. D. Deveson , C. Waters , A. Spessa , D. Lawton , P. Feng , D. L. Liu , Sci. Total Environ. 2019, 668, 947–957. 31018473 10.1016/j.scitotenv.2019.02.439

[108] S. H. Yousef , H. A. Mustafa , L. H. Alwan , Nursing 2022, 22(2), 2500–2504.

[109] M. E. Yuga , P. Wani , J. Appl. Sci. 2022, 10(3), 332–341.

[110] X. Zhao , H. Cui , Y. Wang , C. Sun , B. Cui , Z. Zeng , J. Agric. Food. Chem. 2017, 66(26), 6504–6512. 28654254 10.1021/acs.jafc.7b02004

[111] J. Zhou , Z. Lin , Y. Ju , M. A. Rahim , J. J. Richardson , F. Caruso , Acc. Chem. Res. 2020 a, 53(7), 1269–1278. 32567830 10.1021/acs.accounts.0c00150

[112] J. Zhou , Z. Lin , M. Penna , S. Pan , Y. Ju , S. Li , Y. Han , J. Chen , G. Lin , J. J. Richardson , I. Yarovsky , F. Caruso , Nat. Commun. 2020 b, 11(1), 1–8. 32968077 10.1038/s41467-020-18589-0 PMC7511334

[113] M. Ziaee , S. Moharramipour , A. Mohsenifar , J. Pest Sci. 2014, 87(4), 691–699.

